# Contribution of the Oligomeric State to the Thermostability of Isoenzyme 3 from *Candida rugosa*

**DOI:** 10.3390/microorganisms6040108

**Published:** 2018-10-19

**Authors:** María-Efigenia Álvarez-Cao, Roberto González, María A. Pernas, María Luisa Rúa

**Affiliations:** Department of Food and Analytical Chemistry, Sciences Faculty of Ourense, University of Vigo, As Lagoas s/n, 32004 Ourense, Spain; mariaealvarez@uvigo.es (M.-E.Á.-C.); robergg@uvigo.es (R.G.); mapernas@mundo-r.com (M.A.P.)

**Keywords:** *Candida rugosa*, lipase, kinetic, interfacial activation, inhibition, dimerization, structure

## Abstract

Thermophilic proteins have evolved different strategies to maintain structure and function at high temperatures; they have large, hydrophobic cores, and feature increased electrostatic interactions, with disulfide bonds, salt-bridging, and surface charges. Oligomerization is also recognized as a mechanism for protein stabilization to confer a thermophilic adaptation. Mesophilic proteins are less thermostable than their thermophilic homologs, but oligomerization plays an important role in biological processes on a wide variety of mesophilic enzymes, including thermostabilization. The mesophilic yeast *Candida rugosa* contains a complex family of highly related lipase isoenzymes. Lip3 has been purified and characterized in two oligomeric states, monomer (mLip3) and dimer (dLip3), and crystallized in a dimeric conformation, providing a perfect model for studying the effects of homodimerization on mesophilic enzymes. We studied kinetics and stability at different pHs and temperatures, using the response surface methodology to compare both forms. At the kinetic level, homodimerization expanded Lip3 specificity (serving as a better catalyst on soluble substrates). Indeed, dimerization increased its thermostability by more than 15 °C (maximum temperature for dLip3 was out of the experimental range; >50 °C), and increased the pH stability by nearly one pH unit, demonstrating that oligomerization is a viable strategy for the stabilization of mesophilic enzymes.

## 1. Introduction

Proteins isolated from thermophilic microorganisms, or metagenomes from thermal environments, show high catalytic activity and thermostability at high temperatures. In general, they show high thermostability because they are oligomers that have large hydrophobic cores and increased electrostatic interactions, disulfide bonds, salt-bridging, and surface charges [1]. Oligomerization is recognized as a mechanism for protein stabilization to confer a thermophilic adaptation [2]. Accepting the importance of the oligomers quaternary structure, many authors have studied how to enhance catalytic activity and thermal stability [3]. The strategies followed included site-directed mutagenesis [4,5,6], insertions [7], or deletions of hydrophobic residues that lead to different molecular aggregation states or improve the thermodynamic stability of the oligomeric assembly [8]. Recently, it was observed that mutations located not only inside but also outside the protein interfaces introduce conformational changes that can alter the oligomeric balance (the transition from monomers to oligomers) by creating new bonds, or through the indirect stabilization of protein dynamics, again suggesting a relation between higher-order oligomeric states and thermostability [9,10]. Furthermore, although mesophilic proteins show less thermostability than their thermophilic homologs, it has been observed that protein-protein interactions allow both the homo-oligomeric [11,12] and hetero-oligomeric [13] organization of a wide variety of mesophilic enzymes, which play an important role in many biological pathways and even in cell-cell adhesion processes. As an example, it was recently demonstrated that the reduction in the oligomeric state, through the substitution of conserved amino acid residues, served to abolish the high thermostability of enzymes from pathogenic microorganisms [14]. 

Lipases (triacylglycerol acyl hydrolases EC 3.1.1.3.) constitute a wide family of enzymes with the natural catalytic function of hydrolyzing ester bonds in aqueous media, but they can also catalyze ester synthesis in organic media, having thus an enormous biotechnological potential with regard to several industries [15]. *Candida rugosa* (synonym *Candida cylindracea*) lipases are among the most important lipases from a commercial point of view. They are expressed from a complex family of highly related genes, with at least eight of them identified. Several products of these genes (Lip1–Lip5 and LipJ08) have been biochemically obtained, either using crude commercialized extracts [16,17,18,19], cultures of the wild-type organism [20,21,22], or after heterologous expression of synthetic codon-optimized nucleotide sequences [23,24,25,26,27,28,29,30,31,32,33]. Since *C. rugosa* uses a non-universal codon CUG that codes for the amino-acid serine [34], previous attempts to express the native genes were unsuccessful. These purified isoenzymes differ in terms of amino acid sequence, isoelectric point, and glycosylation degree. An excellent recent revision on the subject can be found in Reference [35]. It is accepted that lipases prefer to act on water-insoluble, long-chain triglycerides, often showing interfacial activation phenomena [36]. An explanation of interfacial activation comes from the presence of an amphiphilic movable flap in the structure of most lipases [37]. In the so-called closed or inactive state, the flap covers the active-site region, avoiding its exposure to the solvent, but in the presence of a lipidic substrate, it displaces, leaving a large hydrophobic area exposed around the active site that contributes to the recognition and binding of substrates [38].

The structural basis for the *C. rugosa* lipase activation came from the Lip1 crystals obtained in both its open and closed conformations [39,40], while Lip2 was crystallized in its closed state [41] and Lip3 in an open dimeric conformation [42,43]. Interestingly, isoenzyme Lip3 is so far the only one within the family for which stable dimers have been also purified and biochemically characterized [21,44], providing a perfect model for studying the effect of homodimerization on the thermostability of a mesophilic enzyme. At the kinetic level, the dimerization of *C. rugosa* Lip3 converts this lipase into a very efficient enzyme to hydrolyze water-soluble esters [21,45], but the effect of non-catalytic hydrophobic interphase has not been previously analyzed. We report the effect of homodimerization on both isoenzyme Lip3 thermostabilization and kinetics.

## 2. Materials and Methods

### 2.1. Materials

Lipase type VII from *C. rugosa* was obtained from Sigma Chemicals Co. (St Louis, MO, USA), tributyrin and triacetin from Fluka (Deisenhofen, Germany), and sodium deoxycholate from Amresco (Solom, OH, USA). Gels for protein purification, DEAE-Sephacel, Phenyl-Sepharose CL-4B, Sephacryl HR 100, and the Sephacryl S200 column were from GE Healthcare (Piscataway, NJ, USA). All chromatographic steps were performed on a fast protein liquid chromatography (FPLC) system (Pharmacia Biotech, Sweden).

### 2.2. Lipases Purification

Dimeric Lip3 was purified as described in Reference [19] and monomeric Lip3 as in Reference [18]. Pure dimeric Lip3 was obtained from the commercial crude powder by means of hydrophobic chromatography followed by size exclusion chromatography using, respectively, Phenyl-Sepharose CL-4B and Sephacryl HR100 gels on lab-mounted columns. To obtain monomeric Lip3, an ethanol precipitation step was implemented before the chromatographic steps, namely anionic-exchange chromatography, using DEAE-Sephacel gel, followed by gel filtration with Sephacryl HR100 gel on lab-mounted columns. A scheme of the Lip3 purification procedure (dimer and monomer) was shown previously [45]. During purification, lipase activity was measured in a pH-stat (Methrom, Switzerland) at 30 °C using tributyrin emulsions stabilized with Arabic gum as described in Reference [46]. The reaction was started by adding enzyme aliquots, which caused the release of the fatty acids esterified to the glycerol moiety. pH was kept constant at 7.0 by automatically adding 0.01 M NaOH for at least 10 min. Initial hydrolysis rates were determined from the slope on the linear part of the obtained plots. One unit (U) is the amount of enzyme that liberates 1 μmol of fatty acid per min under the above conditions.

### 2.3. Size Exclusion Chromatography (SEC)

Purified fractions of dimeric Lip3 were pooled and further purified by SEC using a Sephacryl S200 column equilibrated in 25 mM Tris/HCl buffer (pH 7.5) containing 0.15 M NaCl at 0.3 mL/min. The nature of the interactions responsible for maintaining the homodimers was investigated by SEC as indicated above with the addition of 1% (*w*/*v*) sodium cholate in the running buffer. The high molecular weight marker kit from Sigma was used for calibration. 

### 2.4. Enzyme Kinetic Assays

Purified samples of mLip3 and dLip3 were assayed following the initial hydrolysis rate of triacetin in a pH-stat (Methrom, Switzerland). The assays were performed in 5 mM Tris/HCl buffer (pH 7.0) containing 0.1 M CaCl_2_ at 30 °C, and variable amounts of triacetin (from 35 mM–1.06 M). All assays were done keeping the same stirring speed, while care was taken to avoid the formation of air bubbles in the reaction vessel. The reaction was started with the addition of the enzyme and at least triplicates of each assay were made. One activity unit was defined as the amount of enzyme that released 1 µmol of fatty acids per min. The solubility of triacetin in the reaction conditions was estimated measuring the turbidity as described in Reference [47] and resulted to be 270 mM [21].

### 2.5. Other Methods

Protein concentration was determined by the Lowry method [48], using bovine serum albumin as standard.

### 2.6. Effect of Temperature and pH on the Stability of C. rugosa Isoenzyme 3

Optimization of the temperature (T)-pH stability conditions for mLip3 and dLip3 was obtained using the response surface methodology (RSM). To that end, a 2^2^-central composite orthogonal was designed. The factors were the independent variables studied at five levels (−α, −1, 0, +1, +α) being 0 the central point and α = 1.267 the axial point having for each factor from the center of the experimental domain. The range (pH 5–9 and temperature 30–50 °C) and codification of the variables was as in [22]. The values were coded according to the following equation

*x_i_* = (*X_i_* − *X*_0_)/Δ*X_i_*, (1)

where *x_i_* and *X_i_* are the coded and real values of the independent variable *i*, *X*_0_ is the real value of the independent variable *i* at the central point, and Δ*X_i_* is the step change value. Table 1 shows real and coded values.

Such a design consisted of a set of 13 experiments where the central point was repeated 5 times to estimate the experimental error. Aliquots of pure lipase solutions were incubated for 30 min (or 1 h) in 0.2 M buffers at different pH values and temperatures. The final protein concentration in the mixtures was 0.25 mg/mL. The following buffers were used: Citrate-phosphate buffer (pHs 5.0 and 5.4), sodium phosphate (pH 7.0), and EPPS (*N*-[2-hydroxyethyl]piperazine-*N*’-[3-propane sulfonic acid]) (pHs 8.6 and 9.0). After incubation, the remaining activity was determined in a pH-stat using tributyrin emulsions as described above. Controls consisted of samples of the respective untreated mLip3 and dLip3. The stability of mLip3 and dLip3 was quantified as the percentage of residual activity measured in each condition.

From the experimental data, second-order quadratic models were built to correlate the response with the independent variables or factors, and the optimum point of the empiric model was obtained by solving the regression equation and analyzing the response surface contour plots. The statistical significance of each model was evaluated by analysis of variance (ANOVA) with 95% confidence intervals.

### 2.7. Statistical Analysis

Statistical data treatment was done using the StatGraphics Centurion XVI (Statpoint Technologies, Inc., Warrenton, OR, USA) package and graphics drawing was performed with the programs GraphPad Prism 5 (La Jolla, CA, USA) and SigmaPlot 12.0 (Systat Software, Inc., San Jose, CA, USA). The significance of the data was tested with a Student’s *t*-test and results were considered significant for *p*-values less than or equal to 0.05.

## 3. Results

### 3.1. Purification and Molecular Characterisation

Purification of the monomer (mLip3) and dimer (dLip3) of Lip3 *C. rugosa* isoenzyme from a commercial preparation of *C. rugosa* lipase was done following published procedures. The obtained enzymes showed similar specific activities, determined with tributyrin as the substrate, (1030 U/mg for dLip3 and 1500 U/mg for mLip3) and were within the expected range for the pure forms [21,45]. When monomers were re-chromatographed in SEC columns run with buffered solutions (without sodium cholate), only monomers were detected, and the same was found for the dimers.

**Figure 1:**
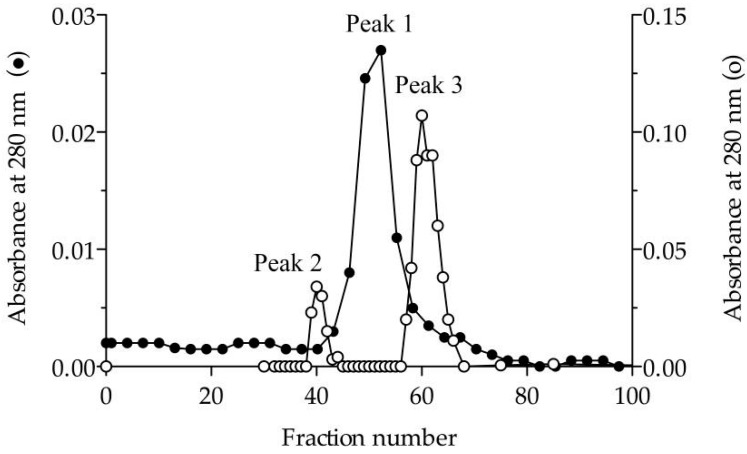
Elution profile in size-exclusion chromatography. Sephacryl S200 column (1.6×60 cm) equilibrated in 25 mM Tris-HCl (pH 7.5) containing 150 mM NaCl in the absence (●) and presence (○) of 1% (*w*/*v*) sodium cholate. Peak 1 corresponds to dimeric Lip3 (117 kDa). Peak 2 and peak 3 correspond to a high molecular weight lipase-cholate aggregate and monomeric Lip3 (60 kDa), respectively. Loaded sample: Dimeric Lip3. Flow: 0.3 mL/min. Fractions: 3 mL.

**Figure 2:**
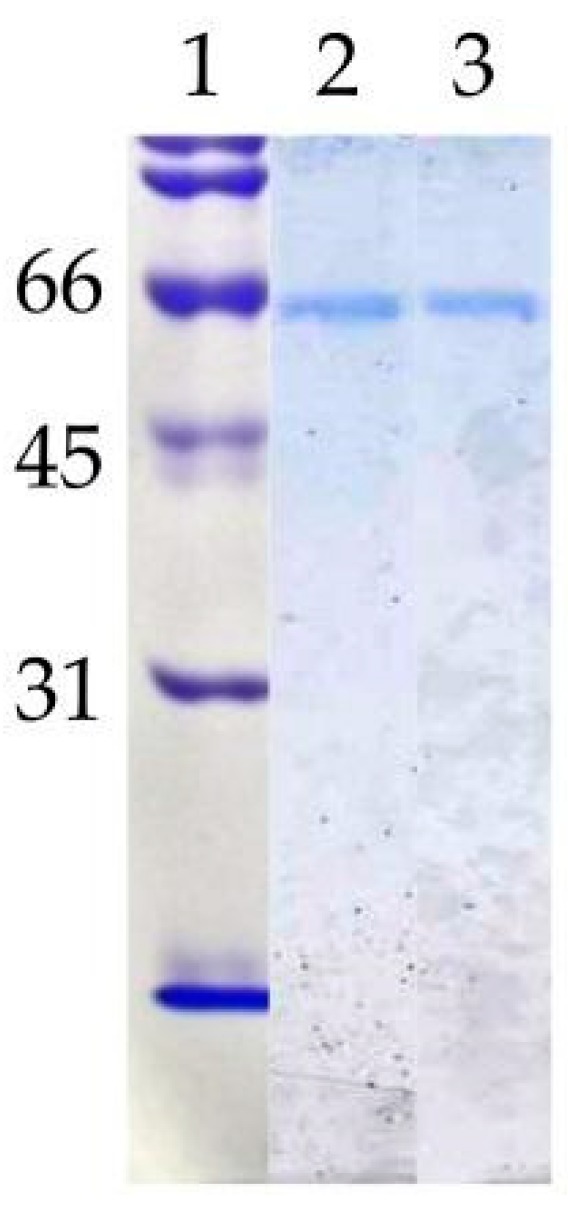
Polyacrylamide gel electrophoresis (SDS-PAGE). Lane 1: Molecular weight standards (kDa); lane 2: mLip 3 (1 µg); lane 3: dLip3 (1 µg). 15% acrylamide gel, Coomassie Blue staining.

To verify the nature of the composition of the dLip3, we performed size-exclusion chromatography (SEC), in the absence or presence of the detergent sodium cholate, and the results are shown in Figure 1. By SEC run without detergent, analyses revealed a single protein fraction with an apparent molecular weight of 117 kDa, which correlates with the expected molecular weight of the dimer [21,45]. When pure Lip3 monomers were chromatographed in the same conditions, only monomers were detected. However, if an aliquot of dLip3 was chromatographed, this time the column being equilibrated with 1% (*w*/*v*) sodium cholate dissolved in equilibration buffer, one high-molecular weight species without activity was eluted in the column void volume, suggesting it could correspond to a high Mw lipase-cholate aggregate. A second protein peak active against tributyrin was eluted in a volume corresponding to 60 kDa (expected for the monomer). In either case, upon 14% SDS-PAGE analysis under reducing conditions, the two tributyrin-active fractions obtained from each run migrated as large homogeneous bands, consistent with the expected Mw of the Lip3 monomer (Figure 2). These results indicate that dimerization of Lip3 molecule is primarily mediated by hydrophobic interactions, which is consistent with the previously reported crystallographic resolution data [21,43]. The hydrophobic nature of the monomer–monomer interaction also explains why the omission of organic solvents and/or detergents during the isolation of the dimer from the commercial impure preparation was crucial [21,45].

### 3.2. Effect of Oligomerization on Enzyme Kinetics

**Figure 3:**
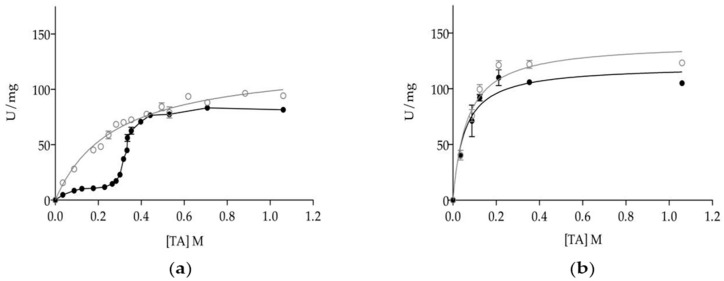
Dependence of specific activity of dLip3 (○) and mLip3 (●) on triacetin concentration in the absence (**a**) and presence (**b**) of 25% (*v*/*v*) hexane. The assays were performed at 30 °C and pH 7.0. Triacetin concentration: From 35 mM–1.06 M. Lipase concentration: 80 µM. Triacetin solubility limit was 270 mM. Data from mLip3 series was reproduced with permission from Pernas et al. [20].

Previous work on the functional characteristics of *C. rugosa* isoenzymes has shown that monomeric isoenzymes Lip1, Lip2 and Lip3 were kinetically distinct to the Lip3 homodimer. However, substrate specificities with triacylglyceride series [18,22] or cholesteryl oleate [38] were different for each isoenzyme, as monomers all showed proper lipase kinetics with interfacial activation once the solubility limit of triacetin (or tributyrin) was overcome [21,38]. Dimerization allows Lip3 enzyme to hydrolyze soluble triacetin (TA) at a high rate, shifting the kinetic model to a *Michaelis-Menten* type [20,21,45]. Kinetics of mLip3 and dLip3 on triacetin are reproduced in Figure 3a to highlight how differences on specific activity tend to minimize above the solubility limit of TA (270 mM) when a substrate interface is formed, subsequently allowing for mLip3 activation.

In this work, we investigated the effect of including a non-catalytic interface in the reaction media together with TA at different concentrations. To that end, the presence of a hydrophobic organic solvent with a high partition coefficient, such as hexane (log *p* = 3.764), was chosen to be included at a concentration of 25% (*v*/*v*), enough to form a two-phase system. Figure 3b shows that in the presence of hexane, the activity profiles for mLip3 and dLip3 were almost indistinguishable and compatible with *Michaelis-Menten* type kinetics. For example, at [TA] = 124 mM (well below the solubility of TA), activity was 10.33 ± 0.82 U/mg (mLip3) and 36.30 ± 1.50 U/mg (dLip3) without hexane, but in reaction media supplemented with hexane, both forms showed a specific activity slightly above 90 U/mg (91.90 ± 1.31 U/mg (mLip3), 99.56 ± 2.15 U/mg (dLip3)). This meant an increase factor of 2.7 for mdLip3, but this was as high as 8.9 for mLip3. 

### 3.3. Effect of Dimerization on Enzyme Thermostability

To investigate the relationship between dimerization and thermostability, we next studied the combined effect of pH and temperature on the stability of mLip3 and dLip3 with the response surface methodology (RSM). The design of the experimental matrix and corresponding results for each experiment are given in Table 2. From the results obtained for each enzyme in the experimental matrix, the quadratic polynomial equations to describe stability were:

mLip3 (30 min): % residual activity = 87 − 31pH − 12T − 27pH^2^ − 11pHT − 6T^2^, (2)

dLip3 (30 min): % residual activity = 89 − 9pH − 8T − 11pH^2^ − 7pHT, (3)

dLip3 (1 h): % residual activity = 89 − 10pH − 7T − 15pH^2^ − 10pHT, (4)

ANOVA tests were performed to validate the quadratic models of the stability of mLip3 and dLip3, testing the statistical significance of each effect by comparing its mean square against an estimated experimental error. As can be seen in the Table 3, linear and quadratic effects for pH and temperature, as well as its interaction (pHT), were statistically significant (*p* < 0.05) to describe mLip3 stability after 30 min of incubation. On the other hand, although the statistically non-significant quadratic effect of temperature (T^2^) of both multiple regression analyses applied to dLip3 was eliminated, the model described at 30 min of incubation showed a significant lack of fit (data not shown), while the model shown at 1 h could be validated and adequately used to describe the stability of dLip3 (Table 4). The *R*^2^ statistic explains the 99.56% and 93.81% of the variability of the response attributed to the independent variables for mLip3 and dLip3, respectively, and both models were appropriated under the experimental conditions (lack of fit-test, *p* > 0.05). 

**Figure 4:**
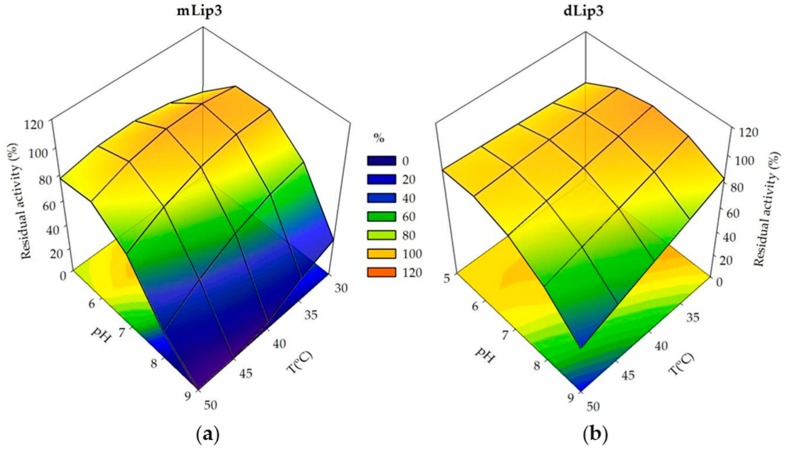
Response surfaces and contour plots generated using experimental design data. (**a**) Residual activity of mLip3 after pH and T treatment for 30 min; (**b**) Residual activity of dLip3 after pH and T treatment for 1 h.

The surface response 3D-plots allowed us to visualize the interactions of the relative thermostability and the acidophilic character of purified mLip3 and dLip3 (Figure 4). Both monomeric and dimeric forms were more sensitive to alkaline pH values than to high temperatures; dLip3 showed the highest thermostability, but the lack of a “T^2^” term in Equation (4) suggests that the optimum for temperature was out of the experimental range (>50 °C). For mLip3, optimum temperature was much lower (35 °C), even though the incubation time was only 30 min compared to 60 min for dLip3. The highest residual activity (maximum stability) was obtained at lower pH values for mLip3 (pH 6.3) than for dLip3 (pH 7.14) (Table 5).

## 4. Discussion

Dimers and oligomers are often the functional form of proteins and may have been evolutionarily selected to confer thermostability on them, particularly in thermophilic microorganisms from both *Archaea* and *Bacteria* domains, as these protein subunit associations can result in an extra stabilizations to cope with extreme temperatures [49,50,51].

In the case of lipases, several have been crystallized in their open conformation without the presence of substrates or inhibitors, suggesting that exposition of hydrophobic areas surrounding the active center occurs in the unbound enzyme [15,52]. The exposed large hydrophobic pocket can promote the association between two open lipases, hence enabling oligomerization [53,54]. Indeed, oligomeric structures appear to be a common feature of the lipases families, and strong correlations between thermostability-aggregation state and catalytic activity-aggregation state have been often described in the literature. For example, non-ionic detergents disaggregate lipases from *Thermosyntropha lipolytica* into less thermostable monomers [55]. The same loss of thermostability was observed for mixtures of dimeric and monomeric forms of lipases in solution under conditions in which disaggregation is promoted (reducing protein concentration and/or including detergents), both in mesophilic and thermophilic microorganisms, such as *Pseudomonas fluorescens* [56], *Alcaligenes* sp. [57], or *Thermomyces lanuginosus* [53]. Mixed results can be found with regard to the effect of aggregation on catalytic activity. For instance, an increase in *P. cepacia* lipase activity was reported as a result of its disaggregation by the addition of 2-propanol [58,59], or *Bacillus thermocatenulatus* lipase (BTL2), treated with some detergents [60], whereas no effect was observed for LipA and LipB from *T. lipolytica* [54]. Still, for *T. lanuginosus,* lipase dimeric forms are less active than monomers [53].

*C. rugosa* Lip3 isoenzyme has not just been purified to homogeneity as a monomer, but also as a stable dimer [21,44,45] and structure of uncomplexed and linoleate-bound dLip3, known at 1.9 Å and 2.0 Å resolution [42,43]. These gave us the opportunity to study directly the effect of dimerization on catalytic activity and stability [43]. The 3D-structure revealed a dimeric association of two Lip3 monomers with their flaps open (active conformation) and the active-site gorges facing each other, thus shielding the hydrophobic environment of the catalytic triads from the aqueous medium. Four openings are generated at the dimer interface (two independent and two symmetry-related) [43]. In a previous paper, we built a model for TA based on the trilaurin structure available in the Cambridge DataBase (code BTRILA) and, using the program X-PLOR [61], the maximum dimension for the TA model was estimated as 8 Å, smaller than the dimensions of the openings (17 × 9 Å and 13 × 8 Å) [21]. The free access of TA through the openings was also confirmed using the O graphic program [62]. Therefore, the openings were large enough to allow the free entrance of triacetin molecules into the active site, without having to dissociate the dimer [21]. This convincingly explains why dimerization allows the observed shift on the dLip3 kinetic model towards a *Michaelis-Menten* type, as opposed to mLip3, which behaves like a proper lipase showing interfacial activation (Figure 3a). These results pinpoint the importance of a hydrophobic interface in lipases catalysis. To deepen exploration into this aspect of catalysis, we carried out a kinetic experiment, including a non-catalytic hydrophobic interface, into the reaction media, together with the substrate TA. Since polar solvents like isopropanol and acetonitrile often tend to decrease the enzyme activity, hexane (high log *p* value) was used. These results showed how dLip3 and mLip3 kinetics on TA were nearly indistinguishable, proving that monomer and dimer were not catalytically different under those conditions. Thus, the monomer flap opening was triggered by a hydrophobic interface even if this was non-catalytic (hexane).

As indicated above, oligomerization is suggested to be an important mechanism for increasing or maintaining the thermostability of proteins [10]. Indeed, for *C. rugosa* Lip3 isoenzyme, dimerization increases by more than 15 °C its thermostability (maximum T for dLip3 was out of the experimental range; >50 °C), well above what was expected for an enzyme from a mesophile such as *C. rugosa*. In addition, the best pH for stability was nearly one-pH unit higher for the homodimer. Although results are difficult to extrapolate due to the different experimental approaches followed, the stability of *C. rugosa* Lip1 isoenzyme was recently improved by rational design methods, with the increase in optimum temperature around 10 °C and the pH optimum remaining unchanged [63]. The extensive protein-protein contacts generated by Lip3 dimerization have therefore resulted in a substantial stabilization. In addition, enzyme inactivation under acidic and alkaline conditions may be caused by the instability of hydrophilic residues on the molecular surface [63,64]. Thus, increasing the hydrophobic interactions can regulate protein pH tolerance, which is also consistent with our results. Based on the premise that oligomerization is a viable evolutionary strategy for protein stabilization, Fraser et al. [10] conducted a laboratory-directed evolution experiment that allowed the selection of a thermostable variant of the αE7 carboxylesterase from *Lucilia cuprina*, with increased levels of dimeric and tetrameric quaternary structures. As opposed to the *C. rugosa* Lip3 here reported, the greatest thermostability was linked to those oligomers with the lowest catalytic activity.

In summary, our work shows that Lip3 functions as a monomer, but homodimerization expands its specificity (a better catalyst on soluble substrates) and improves pH and T stability. For homodimerization to occur, the key event is the activation of two lipase monomers, which expose a large fraction of hydrophobic and aromatic residues on their interfaces that stabilize the complex [21,43]. In our view, the whole process resembles the so-called domain swapping, an accepted mechanism to explain homodimerization [65,66]. Domain swapping includes opening up of the protein monomeric conformation and the exchange of identical regions between the two monomers [11,67]. A number of studies analyzed the properties of interdomain linker regions since they are flexible and might be responsible for domain swapping. Certain residues (especially Pro) in the interdomain linker region might affect the monomer-dimer equilibrium [11,68,69]. Interestingly, within the *C. rugosa* lipase family, the transition between both open and closed conformers is not restricted to a single flap movement. On the contrary, its secondary structure is refolded and accompanied by a *cis*/*trans* isomerization of a Pro92 peptide bond that likely increases the energy required for the transition between the two stages [40]. Therefore, our results highlight oligomerization as a viable strategy for protein stabilization of mesophilic enzymes susceptible to be exploited for biotechnological applications.

## Figures and Tables

**Table 1:** Real and coded values of independent variables in the factorial design 2^2^-central composite.

Real Values	Coded Values ^1^				
	−1.267	−1	0	1	1.267
pH	5	5.4	40	8.6	9
T (°C)	30	32.1	7	47.9	50

^1^*x_i_* = (*X_i_* − *X*_0_)/Δ*X_i_*, *i* = 1, 2.

**Table 2:** Experimental design matrix and comparison of observed data and predicted from RSM.

Run	Coded Values		Real Values		Residual Activity (%) ^1^			
	*x*_1_	*x*_2_	pH	T	mLip3		dLip3	
					Observed	Predicted	Observed	Predicted
1	1	1	8.6	47.9	1.5	−0.09	40.20	46.08
2	1	−1	8.6	32.1	48.3	47.10	82.50	81.24
3	−1	1	5.4	47.9	83	84.83	83.80	88
4	−1	−1	5.4	32.1	84	86.22	84.30	81.36
5	1.267	0	9	40	2.2	4.51	54.90	51.74
6	−1267	0	5	40	86.2	83.10	78.90	78.39
7	0	1.267	7	50	62.9	62.81	88.70	80.18
8	0	−1.267	7	30	94.3	93.60	95.50	98.24
9	0	0	7	40	86.2	87.42	91.40	89.21
10	0	0	7	40	83.6	87.42	85.60	89.21
11	0	0	7	40	90.4	87.42	87.40	89.21
12	0	0	7	40	87.5	87.42	87.00	89.21
13	0	0	7	40	89.1	87.42	91.10	89.21

^1^ The residual activities of mLip3 and dLip3 were measured after 30 min and 1 h of incubation, respectively.

**Table 3:** ANOVA for the response surface quadratic model for the stability of mLip3 after pH and T treatment for 30 min ^1^.

Factor	SS ^2^	DF ^3^	MS ^4^	*F-*Value	*p*-Value ^5^
pH	6935.61	1	6935.61	998.93	0.0000
T	1063.84	1	1063.84	153.23	0.0002
pH^2^	3804.72	1	3804.72	547.99	0.0000
pHT	524.41	1	524.41	75.53	0.0010
T^2^	169.87	1	169.87	24.47	0.0078
Lack of fit	27.74	3	9.25	1.33	0.3817
Pure error	27.77	4	6.94	-	-
Total	12,554.0	12	-	-	-

^1^*R*^2^ = 0.9956. ^2^ Sum of squares. ^3^ Degrees of freedom. ^4^ Mean square. ^5^ *p* ≤ 0.05 denotes a statistically significant difference at the 5% level.

**Table 4:** ANOVA for the response surface quadratic model for the stability of dLip3 after pH and T treatment for 1 h ^1^.

Factor	SS ^2^	DF ^3^	MS ^4^	*F-*Value	*p*-Value ^5^
pH	796.99	1	796.99	117.90	0.0004
T	366.61	1	366.61	54.23	0.0018
pH^2^	1166.01	1	1166.01	172.49	0.0002
pHT	436.81	1	436.81	64.62	0.0013
Lack of fit	155.39	4	38.85	5.75	0.0594
Pure error	27.04	4	6.76	-	-
Total	2948.85	12	-	-	-

^1^*R*^2^ = 0.9381. ^2^ Sum of squares. ^3^ Degrees of freedom. ^4^ Mean square. ^5^ *p* ≤ 0.05 denotes a statistically significant difference at 5% level.

**Table 5:** Optima pH and temperature values for mLip3 and dLip3 stability.

	mLip3	dLip3
pH	6.3	7.14
Temperature (°C)	35	>50

## References

[1] Reed C.J., Lewis H., Trejo E., Winston V., Evilia C. (2013). Protein adaptations in archaeal extremophiles. Archaea.

[2] Giuliani M.C., Tron P., Leroy G., Aubert C., Tauc P., Giudici-Orticoni M.T. (2007). A new sulfurtransferase from the hyperthermophilic bacterium *Aquifex aeolicus*: Being single is not so simple when temperature gets high. FEBS J..

[3] Devenish S.R.A., Gerrard J.A. (2009). The role of quaternary structure in (β/α)8-barrel proteins: Evolutionary happenstance or a higher level of structure-function relationships?. Org. Biomol. Chem..

[4] Linde M., Heyn K., Merkl R., Sterner R., Babinger P. (2018). Hexamerization of geranylgeranylglyceryl phosphate synthase ensures structural integrity and catalytic activity at high temperatures. Biochemistry.

[5] Loveridge E.J., Rodriguez R.J., Swanwick R.S., Allemann R.K. (2009). Effect of dimerization on the stability and catalytic activity of dihydrofolate reductase from the hyperthermophile *Thermotoga maritima*. Biochemistry.

[6] Byun J.-S., Rhee J.-K., Kim N.D., Yoon J., Kim U., Koh E., Oh J.-W., Cho H.-S. (2007). Crystal structure of hyperthermophilic esterase EstE1 and the relationship between its dimerization and thermostability properties. BMC Struct. Biol..

[7] Li P.-Y., Chen X.-L., Ji P., Li C.-Y., Wang P., Zhang Y., Xie B.-B., Qin Q.-L., Su H.-N., Zhou B.-C. (2015). Interdomain hydrophobic interactions modulate the thermostability of microbial esterases from the Hormone-ensitive Lipase family. J. Biol. Chem..

[8] Singh M.K., Shivakumaraswamy S., Gummadi S.N., Manoj N. (2017). Role of an N-terminal extension in stability and catalytic activity of a hyperthermostable α/β hydrolase fold esterase. Protein Eng. Des. Sel..

[9] Perica T., Kondo Y., Tiwari S.P., McLaughlin S.H., Kemplen K.R., Zhang X., Steward A., Reuter N., Clarke J., Teichmann S.A. (2014). Evolution of oligomeric state through allosteric pathways that mimic ligand binding. Science.

[10] Fraser N.J., Liu J., Mabbitt P.D., Correy G.J., Coppin C.W., Lethier M., Perugini M.A., Murphy J.M., Oakeshott J.G., Weik M. (2016). Evolution of protein quaternary structure in response to selective pressure for increased thermostability. J. Mol. Biol..

[11] Nishi H., Hashimoto K., Madej T., Panchenko A.R. (2013). Evolutionary, physicochemical, and functional mechanisms of protein homooligomerization. Prog. Mol. Biol. Transl. Sci..

[12] Elgharbi F., Ben Hlima H., Ameri R., Bejar S., Hmida-sayari A. (2017). A trimeric and thermostable lichenase from *B. pumilus* US570 strain: Biochemical and molecular characterization. Int. J. Biol. Macromol..

[13] Marsh J.A., Teichmann S.A. (2014). Protein flexibility facilitates quaternary structure assembly and evolution. PLoS Biol..

[14] Anand S., Sharma C. (2018). Glycine-rich loop encompassing active site at interface of hexameric *M. tuberculosis* Eis protein contributes to its structural stability and activity. Int. J. Biol. Macromol..

[15] Aschauer P., Rengachari S., Lichtenegger J., Schittmayer M., Padmanabha Das K.M., Mayer N., Breinbauer R., Birner-Gruenberger R., Gruber C.C., Zimmermann R. (2016). Crystal structure of the *Saccharomyces cerevisiae* monoglyceride lipase Yju3p. Biochim. Biophys. Acta-Mol. Cell Biol. Lipids.

[16] Veeraragavan K., Gibbs B.F. (1989). Detection and partial purification of two lipases from *Candida rugosa*. Biotechnol. Lett..

[17] Tomizuka N., Ota Y., Yamada K. (1966). Studies on lipase from *Candida cylindracea*. Agric. Biol. Chem..

[18] Rúa M.L., Díaz-Mauriño T., Fernández V.M., Otero C., Ballesteros A. (1993). Purification and characterization of two distinct lipases from *Candida cylindracea*. BBA-Gen. Subj..

[19] Rúa M.L., Ballesteros A. (1994). Rapid purification of two lipase isoenzymes from *Candida rugosa*. Biotechnol. Tech..

[20] Pernas M.A., Pastrana L., Fuciños P., Rúa M.L. (2009). Regulation of the interfacial activation within the *Candida rugosa* lipase family. J. Phys. Org. Chem..

[21] Pernas M.A., López C., Rúa M.L., Hermoso J. (2001). Influence of the conformational flexibility on the kinetics and dimerisation process of two *Candida rugosa* lipase isoenzymes. FEBS Lett..

[22] López N., Pernas M.A., Pas trana L.M., Sánchez A., Valero F., Rúa M.L. (2004). Reactivity of pure *Candida rugosa* lipase isoenzymes (Lip1, Lip2, and Lip3) in aqueous and organic media. Influence of the isoenzymatic profile on the lipase performance in organic media. Biotechnol. Prog..

[23] Chang S.W., Shieh C.J., Lee G.C., Shaw J.F. (2005). Multiple mutagenesis of the *Candida rugosa LIP1* gene and optimum production of recombinant *LIP1* expressed in *Pichia pastoris*. Appl. Microbiol. Biotechnol..

[24] Chang S.-W., Lee G.-C., Shaw J.-F. (2006). Codon optimization of *Candida rugosa LIP1* gene for improving expression in *Pichia pastoris* and biochemical characterization of the purified recombinant Lip1 lipase. J. Agric. Food Chem..

[25] Zhao W., Wang J., Deng R., Wang X. (2008). Scale-up fermentation of recombinant *Candida rugosa* lipase expressed in *Pichia pastoris* using the *GAP* promoter. J. Ind. Microbiol. Biotechnol..

[26] Chang S.-W., Lee G.-C., Shaw J.-F. (2006). Efficient production of active recombinant *Candida rugosa* Lip3 lipase in *Pichia pastoris* and biochemical characterization of the purified enzyme. J. Agric. Food Chem..

[27] Ferrer P., Alarcón M., Ramón R., Dolors Benaiges M., Valero F. (2009). Recombinant *Candida rugosa LIP2* expression in *Pichia pastoris* under the control of the *AOX1* promoter. Biochem. Eng. J..

[28] Lee G.-C., Lee L.-C., Sava V., Shaw J.-F. (2002). Multiple mutagenesis of non-universal serine codons of the *Candida rugosa LIP2* gene and biochemical characterization of purified recombinant Lip2 lipase overexpressed in *Pichia pastoris*. Biochem. J..

[29] Lee L.C., Chen Y.T., Yen C.C., Chiang T.C.-Y., Tang S.-J., Lee G.-C., Shaw J.-F. (2007). Altering the substrate specificity of *Candida rugosa* Lip4 by engineering the substrate-binding sites. J. Agric. Food Chem..

[30] Lee L.-C., Yen C.-C., Malmis C.C., Chen L.-F., Chen J.-C., Lee G.-C., Shaw J.-F. (2011). Characterization of codon-optimized recombinant *Candida rugosa* Lipase 5 (Lip5). J. Agric. Food Chem..

[31] Tang S.-J., Sun K.-H., Sun G.-H., Chang T.-Y., Lee G.-C. (2000). Recombinant expression of the *Candida rugosa* Lip4 lipase in *Escherichia coli*. Protein Expr. Purif..

[32] Tang S.-J., Shaw J.-F., Sun K.-H., Sun G.-H., Chang T.-Y., Lin C.-K., Lo Y.-C., Lee G.-C. (2001). Recombinant expression and characterization of the *Candida rugosa* Lip4 lipase in *Pichia pastoris*: Comparison of glycosylation, activity, and stability. Arch. Biochem. Biophys..

[33] Yen C.-C., Malmis C.C., Lee G.-C., Lee L.-C., Shaw J.-F. (2010). Site-specific saturation mutagenesis on residues 132 and 450 of *Candida rugosa* Lip2 enhances catalytic efficiency and alters substrate specificity in various chain lengths of triglycerides and esters. J. Agric. Food Chem..

[34] Kawaguchi Y., Honda H., Taniguchi-Morimura J., Iwasaki S. (1989). The codon CUG is read as serine in an asporogenic yeast *Candida cylindracea*. Nature.

[35] Barriuso J., Vaquero M.E., Prieto A., Martínez M.J. (2016). Structural traits and catalytic versatility of the lipases from the *Candida rugosa*-like family: A review. Biotechnol. Adv..

[36] Verger R. (1980). Enzyme kinetics of lipolysis. Methods in Enzymology.

[37] Schrag J.D., Cygler M. (1997). Lipases and alpha/beta hydrolase fold. Methods Enzymol..

[38] Mancheño J.M., Pernas M.A., Martínez M.J., Rúa M.L., Hermoso J.A. (2003). Structural insights into the lipase/esterase behavior in the *Candida rugosa* lipases family: Crystal structure of the Lipase 2 isoenzyme at 1.97 Å resolution. J. Mol. Biol..

[39] Grochulski P., Li Y., Schrag J.D., Bouthillier F., Smith P., Harrison D., Rubin B., Cygler M. (1993). Insights into interfacial activation from an open structure of *Candida rugosa* lipase. J. Biol. Chem..

[40] Grochulski P., Li Y., Schrag J.D., Cygler M. (1994). Two conformational states of *Candida rugosa* lipase. Protein Sci..

[41] Mancheño J.M., Pernas M.A., Rúa M.L., Hermoso J.A. (2003). Crystallization and preliminary X-ray diffraction studies of two different crystal forms of the Lipase 2 isoform from the yeast *Candida rugosa*. Acta Crystallogr. Sect. D Biol. Crystallogr..

[42] Pletnev V., Addlagatta A., Wawrzak Z., Duax W. (2003). Three-dimensional structure of homodimeric cholesterol esterase-ligand complex at 1.4 Å resolution. Acta Crystallogr.-Sect. D Biol. Crystallogr..

[43] Ghosh D., Wawrzak Z., Pletnev V.Z., Li N., Kaiser R., Pangborn W., Jornvall H., Erman M., Duax W.L. (1995). Structure of uncomplexed and linoleate-bound *Candida cylindracea* cholesterol esterase. Structure.

[44] Kaiser R., Erman M., Duax W.L., Ghosh D., Jörnvall H. (1994). Monomeric and dimeric forms of cholesterol esterase from *Candida cylindracea*. Primary structure, identity in peptide patterns, and additional microheterogeneity. FEBS Lett..

[45] Pernas M., López C., Prada A., Hermoso J., Rúa M.L. (2002). Structural basis for the kinetics of *Candida rugosa* Lip1 and Lip3 isoenzymes. Colloid Surf. B Biointerfaces.

[46] Pernas M.A., López C., Pastrana L., Rúa M.L. (2000). Purification and characterization of Lip2 and Lip3 isoenzymes from a *Candida rugosa* pilot-plant scale fed-batch fermentation. J. Biotechnol..

[47] Ferrato F., Carriere F., Sarda L., Verger R. (1997). A critical reevaluation of the phenomenon of interfacial activation. Methods Enzymol..

[48] Randall R.J., Lewis A. (1951). Protein measurement with the folin phenol reagent. Readings.

[49] Varejão N., De-Andrade R.A., Almeida R.V., Anobom C.D., Foguel D., Reverter D. (2018). Structural mechanism for the temperature-dependent activation of the hyperthermophilic Pf2001 esterase. Structure.

[50] Robinson-Rechavi M., Alibés A., Godzik A. (2006). Contribution of electrostatic interactions, compactness and quaternary structure to protein thermostability: Lessons from structural genomics of *Thermotoga maritima*. J. Mol. Biol..

[51] Walden H., Bell G.S., Russell R.J.M., Siebers B., Hensel R., Taylor G.L. (2001). Tiny TIM: A small, tetrameric, hyperthermostable triosephosphate isomerase. J. Mol. Biol..

[52] Skjold-Jørgensen J., Vind J., Moroz O.V., Blagova E., Bhatia V.K., Svendsen A., Wilson K.S., Bjerrum M.J. (2017). Controlled lid-opening in *Thermomyces lanuginosus* lipase–An engineered switch for studying lipase function. Biochim. Biophys. Acta-Proteins Proteom..

[53] Gonçalves K.M., Barbosa L.R.S., Lima L.M.T.R., Cortines J.R., Kalume D.E., Leal I.C.R., Mariz e Miranda L.S., De Souza R.O.M., Cordeiro Y. (2014). Conformational dissection of *Thermomyces lanuginosus* lipase in solution. Biophys. Chem..

[54] Madsen J.K., Sørensen T.R., Kaspersen J.D., Silow M.B., Vind J., Pedersen J.S., Svendsen A., Otzen D.E. (2015). Promoting protein self-association in non-glycosylated *Thermomyces lanuginosus* lipase based on crystal lattice contacts. Biochim. Biophys. Acta-Proteins Proteom..

[55] Salameh M.A., Wiegel J. (2010). Effects of detergents on activity, thermostability and aggregation of two alkalithermophilic lipases from *Thermosyntropha lipolytica*. Open Biochem. J..

[56] Fernández-Lorente G., Palomo J.M., Fuentes M., Mateo C., Guisán J.M., Fernández-Lafuente R. (2003). Self-assembly of *Pseudomonas fluorescens* lipase into bimolecular aggregates dramatically affects. Biotechnol. Bioeng..

[57] Wilson L., Palomo J.M., Fernández-Lorente G., Illanes A., Guisán J.M., Fernández-Lafuente R. (2006). Effect of lipase–lipase interactions in the activity, stability and specificity of a lipase from *Alcaligenes* sp.. Enzyme Microb. Technol..

[58] Otzen D.E. (2002). Protein unfolding in detergents: Effect of micelle structure, ionic strength, pH, and temperature. Biophys. J..

[59] Dünhaupt A., Lang S., Wagner F. (1992). Pseudomonas cepacia lipase: Studies on aggregation, purification and on the cleavage of olive oil. Biotechnol. Lett..

[60] Luisa Rúa M., Schmidt-Dannert C., Wahl S., Sprauer A., Schmid R.D. (1997). Thermoalkalophilic lipase of *Bacillus thermocatenulatus*. Large-scale production, purification and properties: Aggregation behaviour and its effect on activity. J. Biotechnol..

[61] Brunger A.T. (1992). X-PLOR, version 3.1: A System for X-ray Crystallography and NMR.

[62] Jones T.A., Zou J.Y., Cowan S.W., Kjeldgaard M. (1991). Improved methods for binding protein models in electron density maps and the location of errors in these models. Acta Crystallogr. A.

[63] Li G., Chen Y., Fang X., Su F., Xu L., Yan Y. (2018). Identification of a hot-spot to enhance: *Candida rugosa* lipase thermostability by rational design methods. RSC Adv..

[64] Oguchi Y., Maeda H., Abe K., Nakajima T., Uchida T., Yamagata Y. (2006). Hydrophobic interactions between the secondary structures on the molecular surface reinforce the alkaline stability of serine protease. Biotechnol. Lett..

[65] Gronenborn A.M. (2009). Protein acrobatics in pairs–Dimerization via domain swapping. Curr. Opin. Struct. Biol..

[66] Bennett M.J., Choe S., Eisenberg D. (1994). Domain swapping: Entangling alliances between proteins. Proc. Natl. Acad. Sci. USA.

[67] Hashimoto K., Nishi H., Bryant S., Panchenko A.R. (2011). Caught in self-interaction: Evolutionary and functional mechanisms of protein homooligomerization. Phys. Biol..

[68] Hashimoto K., Panchenko A.R. (2010). Mechanisms of protein oligomerization, the critical role of insertions and deletions in maintaining different oligomeric states. Proc. Natl. Acad. Sci. USA.

[69] Rousseau F., Schymkowitz J.W.H., Wilkinson H.R., Itzhaki L.S. (2001). Three-dimensional domain swapping in p13suc1 occurs in the unfolded state and is controlled by conserved proline residues. Proc. Natl. Acad. Sci. USA.

